# Relationship Between the Morphology of Oral Membranous Substances and Oral Wetness in Older Individuals Undergoing Long‐Term Tube Feeding in Japan

**DOI:** 10.1111/scd.70078

**Published:** 2025-08-18

**Authors:** Hironao Asahina, Yoshiyuki Okada, Ryo Nishino, Kahoru Todoroki, Kohei Matsumura, Yusuke Yamagami, Tadashi Ogasawara

**Affiliations:** ^1^ Department of Special Care Dentistry Hiroshima University Hospital Hiroshima Japan; ^2^ Department of Dentistry Todoroki Medical Hospital Nagano Japan; ^3^ Matsumura Dental Clinic Osaka Japan; ^4^ Department of Special Care Dentistry Matsumoto Dental University Nagano Japan; ^5^ Matsumoto Dental University Nagano Japan; ^6^ Yokosuna Dental Clinic Shizuoka Japan

**Keywords:** dental care for disabled, dental care for older adults, oral health, oral hygiene, tube feeding, xerostomia

## Abstract

**Aims:**

Oral membranous substances in tube‐fed patients undergo temporal morphological changes, increasing the risk of asphyxiation. While xerostomia is hypothesized to influence these alterations, its precise relationship with oral wetness and specific morphological stages remains unclear. This study aimed to investigate oral wetness as an indicator of xerostomia and predictor of membranous substance formation.

**Methods and Results:**

This study included 18 older tube‐fed patients. Following initial oral care, comprising tooth surface and oral mucosa cleaning, we measured tongue dorsum wetness at 3, 6, 12, 24, and 48 h post‐care. Concurrent visual examinations assessed the presence and morphology of palatal membranous substances, categorizing them into four types: none, mucous, viscous, and dry. We analyzed the relationships between morphology, wetness, and elapsed time. Oral wetness levels varied significantly across morphological types (*p* < 0.001), with dry membranous substances exhibiting the lowest wetness values. Membranous substance morphology significantly correlated with elapsed time (*r* = 0.513, *p* < 0.001) and oral wetness (*r* = −0.390, *p* < 0.001).

**Conclusions:**

Both temporal progression and xerostomia significantly contribute to the formation and morphological evolution of membranous substances. Timely implementation of oral care and moisturization appears crucial for preventing membranous substance formation and mitigating the associated risks.

## 1 Introduction

In Japan, the implementation rate of tube feeding for older adults requiring long‐term care is approximately 60% in long‐term care beds [1], representing its widespread use. Tube‐fed older patients often have compromised oral environments [2] compared to those maintaining oral intake, frequently presenting with oral membranous substances on the palatal mucosa [3]. These substances pose significant risks if they thicken and enter the larynx, potentially causing dysphagia [4], dysarthria [5], airway obstruction [6], and asphyxia [7]. Additionally, they are associated with fever [8] and pneumonia [2], as oral bacteria can migrate to the respiratory tract through the dislodged membranous substances [9, 10]. Thus, preventing these membranous formations is critical. The formation of membranous substances is caused by xerostomia [3]. While monitoring xerostomia in tube‐fed patients may aid in preventing membranous substances, no established thresholds or indicators currently exist for xerostomia‐induced membranous formation.

Current methods for assessing xerostomia include quantitative measurements of salivary flow through various diagnostic tests, such as the chewing gum test, Saxon test, and spitting method, as well as the evaluation of salivary gland function via scintigraphy. However, these conventional methods primarily assess the salivary glands’ secretory capacity rather than directly measuring xerostomia. Furthermore, these testing procedures present significant challenges and may be unsuitable for older patients requiring tube feeding. Alternatively, a novel diagnostic approach has been developed to evaluate oral wetness by measuring the impedance value of the tongue dorsal moisture content using an electrostatic capacitance sensor based on resonance frequency interactions [11]. This methodology enables the efficient assessment of xerostomia even in older patients dependent on tube feeding. Despite these advances, the correlation between oral wetness and membranous substances obtained using this method remains unexplored. Membranous substances undergo temporal morphological transitions from mucous to viscous and ultimately dry forms [12], with xerostomia hypothetically influencing these changes. However, the precise relationship between oral wetness and specific morphological stages remains unclear. This study aimed to investigate these relationships by measuring oral wetness and evaluating temporal changes in palatal membranous substance morphology. Additionally, we sought to establish oral wetness cut‐off values associated with the formation of each morphological stage.

## 2 Materials and Methods

### 2.1 Study Participants

This study was conducted between February and October 2023 at a hospital in Nagano Prefecture, Japan. A total of 30 participants were prescreened, and 18 were included in this study. Inclusion criteria were individuals aged ≥65 years, those in need of long‐term nursing care, and those who were tube‐fed (nasogastric feeding or percutaneous endoscopic gastrostomy tube feeding) without oral intake. Exclusion criteria followed the guidelines for safety management published by the Japanese Association of Rehabilitation Medicine [13], with minor modifications to the heart rate, blood pressure, body temperature, and extrasystole to accommodate bedridden individuals. Patients with pneumonia, cold symptoms, strong refusal behavior, and those fitted with a heated humidifier were excluded. Additionally, patients with Sjögren's syndrome, SNOX (sialadenitis, nodal osteoarthritis, xerostomia) syndrome [14], or autoimmune diseases were excluded (Table 1). Written informed consent was obtained from all participants through their substitutes. A comprehensive overview of the recruitment process is presented in a supplementary flowchart (Figure S1). This study was approved by xxx (approval number: xxx).

**TABLE 1:** Exclusion criteria.

Characteristics	Criteria
Heart rate	More than 100 bpm
Blood pressure	More than 180/110 mmHg
Blood oxygen (SpO_2_)	Less than 90%
Extrasystole	Five times per minute
Body temperature	Over 37.5°C
Clinical findings	Pneumonia with a cold Cough Strong refusal behavior
Heated humidifier	Using
Associated comorbidities	Sjögren's syndrome SNOX syndrome Autoimmune diseases

*Source*: The exclusion criteria were based on the “Guidelines for Safety Management and Promotion in Rehabilitation Medicine,” [13] with some modifications.

### 2.2 Characteristics of Participants

We recorded the participants’ sex, age, degree of bedridden status (the degree of independent living for older people with disabilities, as defined by the Japanese Ministry of Health, Labour and Welfare, 1991), presence or absence of polypharmacy, and underlying medical conditions from hospitalization and admission records. Polypharmacy was defined as the use of more than five prescribed medications [15]. The nurse‐in‐charge confirmed each patient's level of consciousness (Japan Coma Scale), ability to communicate (i.e., whether they could follow instructions to open their mouth), and use of oral moisturizers.

### 2.3 Protocol

**FIGURE 1:**
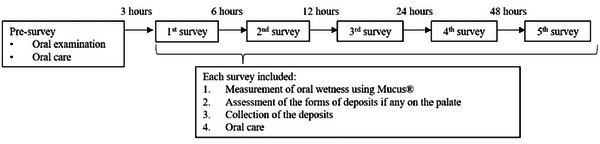
Flow diagram. First, an oral examination was conducted, followed by oral care (presurvey). The first survey was conducted 3 h after oral care at presurvey. The second survey was conducted 6 h after the first survey, and subsequent surveys were repeated 12, 24, and 48 h after the last survey. In each survey, we measured the oral wetness of the tongue dorsum. If deposits were observed on the palate, we recorded their morphology and collected them. Oral care was provided at the end of each survey.

Previously, nurses provided oral care to participants twice daily; however, this care was discontinued for the study. During the presurvey, an oral examination was performed to assess the number of teeth and the presence or absence of constant mouth opening with the participant in the supine position. The participant's mouth was classified as “Constant mouth opening” if it remained open by at least one fingerbreadth without lip stimulation, following a study by Nozawa [16]. After the oral examination, oral care was performed using a toothbrush (Ruscello Grappo P‐30, GC Co., Ltd., Tokyo, Japan), soft‐bristle toothbrush (Homecare toothbrush A‐US, Nagayama Co., Ltd., Osaka, Japan), sponge brush (Hamingood, Molten Corporation, Hiroshima, Japan, and gauze to clean the tooth surfaces and oral mucosa with water. Three hours after the presurvey oral care, oral wetness was measured using Mucus (Life Co., Ltd., Saitama, Japan) at the center of the lingual mucosa, approximately 10 mm from the tip of the tongue, to assess oral dryness. Wetness was defined as dry mouth (≤27.9), borderline dry mouth (28.0–29.5), and normal (≥29.6) [11]. Oral care was then repeated, and oral wetness was reassessed 6–48 h after the oral care, according to the study protocol (Figure 1). At each elapsed time, if deposits were observed on the palatal mucosa, they were collected using tweezers, and their forms were recorded. The collected deposits were soaked in a 10% neutral buffered formalin solution. All oral surveys, oral care, and sample collection were performed by a dentist with over 7 years of experience, certified by the Japanese Society of Gerodontology. The oral examination was performed in an environmentally controlled room with a temperature and relative humidity of approximately 25°C and 30%, respectively. The assessment of oral wetness was avoided during nutritional infusions and was performed only after confirming that the patient was at rest.

### 2.4 Diagnosis of Membranous Substances

The fixed deposit samples were embedded in paraffin, and 4.5‐µm‐thick tissue sections were stained with hematoxylin and eosin. Tissue sections were immersed in xylene, followed by graded ethanol (100%–70%), and washed with tap water at room temperature. The sections were then immersed in hematoxylin for 5 min, rinsed thoroughly, treated with eosin for 5 min, and dehydrated in graded ethanol (70%–100%) and xylene. After staining, the samples were mounted on slides with glass coverslips. Samples were diagnosed as membranous substances only when keratin derived from the stratified squamous epithelium was observed microscopically [3].

**FIGURE 2:**
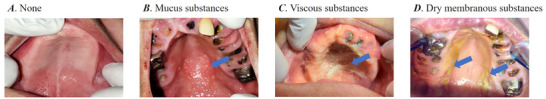
Intraoral photographs. Membranous substances (arrows) show different morphologies and colors from patient to patient. (A) “None” indicates no deposits on the palate, (B) mucous substances (clear or translucent mucus), (C) viscous substances (yellowish‐white or yellow deposits), and (D) dry membranous substances (yellowish‐white or yellow, dry, and non‐viscous deposits.

The morphologies of the membranous substances were classified into four types by visual examination [12]: (1) none (no attachments present); (2) mucous substances (clear or translucent mucus); (3) viscous substances (yellowish‐white or yellow viscous deposits); and (4) dry membranous substances (yellowish‐white or yellow, dry, and non‐viscous deposits) (Figure 2). When two or more shapes were present, the more severe shape was selected.

### 2.5 Statistical Analyses

The Shapiro–Wilk normality test was used to assess whether oral wetness was normally distributed, and the Friedman test was used to analyze changes in the morphologies of each membranous substance and oral wetness over time. The Kruskal–Wallis test was used to compare oral wetness across the four membranous substance morphologies. The Bonferroni and Steel–Dwass methods were used as post hoc tests after the Friedman and Kruskal–Wallis tests, respectively. Spearman's correlation coefficients were calculated to examine the relationship between the membranous substance morphology and quantitative data over time (oral wetness and elapsed time). The cut‐off values for each morphology were examined using receiver operating characteristic (ROC) curve analysis. The area under the curve (AUC) was calculated, and the point on the ROC curve with the minimum distance from the upper left corner of the unit square was used as the cut‐off value. Each test was performed using EZR (version 1.55; Jichi Medical University, Saitama, Japan) [17]. Statistical significance was set at *p* < 0.05.

## 3 Result

### 3.1 Participants’ Physical Characteristics

There were 9 males and 9 females with a mean age of 81.7 ± 9.9 years. All patients were grade C bedridden; 13 had impaired consciousness of JCS II or higher, and 15 had consistently open mouths (Table 2).

**TABLE 2:** Participant characteristics (*n* = 18).

	(*n*)	(%)
**Demographic and general health status**		
*Age, years, mean* (SD)	81.7 (9.9)	
*Male, n* (%)	9 (50.0)	
*Bedridden level*		
J Rank	0	0.0
A Rank	0	0.0
B Rank	0	0.0
C Rank	18	100
*Japan coma scale*		
I	5	27.8
II	10	55.6
III	3	16.7
*Communication*		
Able	2	11.1
Unable	16	88.9
*Polypharmacy*		
No	11	61.1
Yes	7	38.9
**Oral health status**		
*Number of teeth present, mean* (SD)	10.4 (7.6)	
*Constant mouth opening*		
No	3	16.7
Yes	15	83.3
*Oral moisturizers*		
Used	5	27.8
Not used	13	72.2

*Note*: Bedridden levels: J Rank: Have some disability, but are almost independent in daily life and can get out of their home without assistance; A Rank: Almost independent for indoor daily life, but cannot go outside without care; B Rank: Require some care for indoor daily life and mostly stay in bed, but can keep a sitting position; C Rank: Always stay in bed and require care for toileting, eating, and changing clothes. Japan Coma Scale I: the patient is awake without any stimuli; II: the patient can be aroused (then reverts to the previous state after stimulation cessation); and III: the patient cannot be aroused with any forceful mechanical stimuli. Communication: Able to follow mouth‐opening instructions, unable to follow mouth‐opening instructions. Polypharmacy: Using more than five prescribed medicines. Constant mouth opening: Resting mouth opening is always greater than the fingerbreadth.

Abbreviation: SD, standard deviation.

### 3.2 Time Course of Changes in the Oral Membranous Substances and Oral Wetness

**FIGURE 3:**
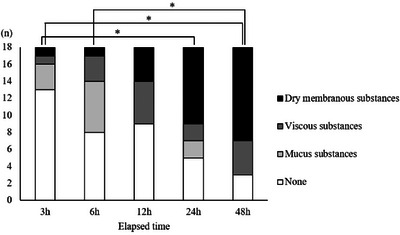
Changes in morphology of membranous substances according to elapsed time. Dry membranous substances increased over time (*p* < 0.001). ^*^ *p* < 0.05.

**FIGURE 4:**
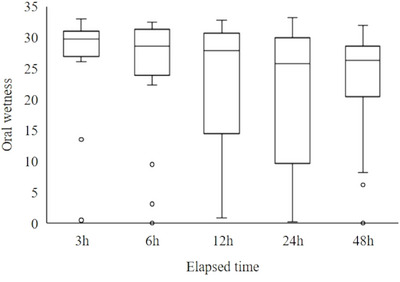
Changes in oral wetness according to elapsed time. Oral wetness decreased over time (*p* = 0.012).

The morphology of the membranous substances and the oral wetness over time are illustrated in Figures 3 and 4. Mucous substances began to form mainly at 3–6 h, viscous substances at 6–12 h, and dry substances at 12–24 h. The morphology of the membranous substances progressed significantly over time (*p* < 0.001). Oral wetness also decreased significantly over time (*p* = 0.012), with oral dryness observed after 12 h (median wetness: 27.9), 24 h (median: 25.8), and 48 h (median: 26.4).

### 3.3 Relationship Between the Morphology of Oral Membranous Substances and Wetness

**FIGURE 5:**
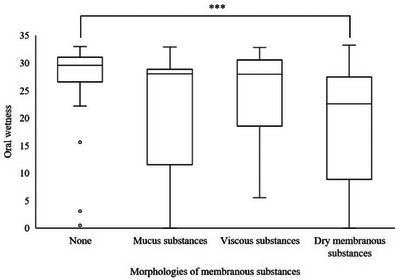
Relationship between oral wetness and morphology of membranous substances. Oral wetness decreased as the morphology deteriorated (*p* < 0.001). ^***^ *p* < 0.001.

The median oral wetness of each membranous substance morphology was 29.6 for none, 28.0 for mucous substances, 27.9 for viscous substances, and 22.6 for dry membranous substances, indicating that the mouth was particularly dry when dry membranous substances were formed. Oral wetness differed significantly by morphology (*p* < 0.001), with a significant difference in the wetness between dry membranous substances and none (*p* < 0.001) (Figure 5).

A significant correlation was observed between membranous substance morphology and oral wetness (*r* = −0.390, *p* < 0.001), as well as elapsed time (*r* = 0.501, *p* < 0.001) (Figures S2, S3).

The cut‐off values for the membranous substance formation were 29.1 (sensitivity: 81.8%, specificity: 57.9%, AUC: 0.694), 28.0 (sensitivity: 60.0%, specificity: 63.2%, AUC: 0.579), and 27.5 (sensitivity: 76.9%, specificity: 68.4%, AUC: 0.790) for mucous, viscous, and dry membranous substances, respectively (Table 3).

**TABLE 3:** Cut‐off values for the formation of each membranous substance morphology.

None vs.	Cut‐off value	Sensitivity	Specificity	AUC	95% CI
Mucous substances	29.1	81.8 %	57.9 %	0.694	0.502–0.886
Viscous substances	28.0	60.0 %	63.2 %	0.574	0.388–0.760
Dry membranous substances	27.5	76.9 %	68.4 %	0.790	0.674–0.906

Abbreviation: CI, confidence interval.

## 4 Discussion

In this study, we examined the relationship between the morphology of membranous substances and oral wetness. Our findings revealed a progressive decline in oral wetness and a significant correlation between oral wetness and membranous substance morphology.

Unlike sputum and crusts, the membranous substances primarily comprise keratin from stratified squamous epithelium [18] and mucin from palatine glands [4, 19], accompanied by inflammatory cells and bacteria [18]. The morphology of these substances evolves temporally, transitioning from mucous to viscous and ultimately to dry membranous substances [12]. Mucous substances exhibit low epithelial cell levels and high mucin content, whereas dry membranous substances demonstrate high epithelial cell concentrations and reduced mucin levels. This progression suggests a shortage of salivary gland secretions and a chronological reduction in the water content of membranous substances. Water reduction can be attributed to xerostomia‐induced evaporation. Parenteral intake is a critical prerequisite for membranous substance formation, with oral dryness and constant mouth opening as primary contributing factors [3]. In tube‐fed older patients with impaired consciousness, reduced tongue mobility significantly compromises the adherence of saliva to the oral mucosa. Simultaneously, constant mouth opening exposes the oral mucosa to ambient air, resulting in xerostomia [20]. In this study, over 72% of participants had consciousness impairment, and over 83% had constant mouth opening. Consequently, we inferred that oral wetness decreased over time because of evaporation. As the mouth dried out, the water content of the membranous substances also decreased, leading to morphological changes. Additionally, the membranous substance morphology demonstrated a stronger temporal correlation than a direct association with oral wetness, suggesting time‐dependent morphological changes, with xerostomia serving as a modifying factor. These findings underscore the importance of timely oral care and strategic mouth moisturization to prevent membranous substance formation, highlighting the need for proactive intervention strategies in patient management.

The calculated cut‐off values for membranous substance formation were 29.1 for mucous substances, 28.0 for viscous substances, and 27.5 for dry membranous substances. Analysis of the mucous substance formation threshold revealed that while a cut‐off value of 29.1 for oral wetness demonstrated high sensitivity (81.8%), it showed relatively low specificity (57.9%). This finding indicates that mucous formation occurred in approximately 40% of cases, even when oral wetness exceeded the threshold value of 29.1. The AUC of 0.694 suggests moderate diagnostic accuracy. Similarly, for viscous substance formation, a cut‐off value of 28.0 yielded sensitivity and specificity values of 60.0% and 63.2%, respectively, with a relatively low AUC of 0.579, indicating suboptimal diagnostic accuracy. In contrast, oral wetness measurements showed significant differences between the dry membranous substance formation group and the non‐group, suggesting that patients developing dry membranous substances experienced more severe oral dryness than those with other morphological types. Notably, the cut‐off value for dry membranous substance formation demonstrated superior diagnostic characteristics, with well‐balanced sensitivity (76.9%) and specificity (68.4%) compared to other morphological types and an improved AUC of 0.790. This AUC value is comparable to that of the Revised Cardiac Risk Index, an established scoring system for predicting perioperative cardiovascular complications in non‐cardiac surgery [21]. These findings provide valuable data for preventing membranous substance formation and serve as a meaningful indicator for implementing oral care protocols in tube‐fed patients.

Matsumura et al. [12] recommend oral care intervals of 6–12 h for tube‐fed patients. This recommendation stems from observations that dry membranous substances, which present significant removal challenges, typically develop within 12–24 h following oral care procedures [12]. These dry membranous substances not only substantially increase the duration of oral care compared to other forms [12] but also pose risks of bleeding during removal [22]. Moreover, they represent significant risk factors for multiple complications, including dysphagia [5], airway obstruction [6], suffocation [7], and respiratory tract infections [9, 10], necessitating particular attention to their prevention. Our survey findings corroborate those of previous research, indicating that maintaining oral care intervals of 6–12 h effectively prevents the formation of dry membranous substances. Additionally, regular monitoring of patients' oral wetness levels and adjusting oral care intervals to maintain measurements above established cut‐off values are crucial. This individualized approach to oral care timing may enhance the efficacy of preventive strategies against dry membranous substance formation.

### 4.1 Limitations and Perspectives

A primary limitation of this study was its small sample size. Participant recruitment was challenging because the study population was restricted to older adults requiring tube feeding. Consequently, the limited sample size may have affected the accuracy of the established cut‐off values for mucous and viscous substance formations.

Additionally, as an observational study, it remains unclear whether maintaining oral wetness above the identified cut‐off values effectively prevents membranous substance formation. Future research should address these limitations through large‐scale studies to enhance the accuracy of the data. Interventional studies are warranted to establish evidence‐based protocols for preventing membranous substance formation.

## 5 Conclusion

Our findings demonstrate that membranous substances undergo temporal morphological transitions, progressing from mucous to viscous and ultimately to dry forms, each associated with distinct oral wetness levels. The progression of these changes is influenced by both elapsed time and xerostomia, suggesting that the timely implementation of oral care and moisturization procedures is crucial for preventing membranous substance formation.

The analysis determined cut‐off values for membranous substance formation at 29.1 for mucous, 28.0 for viscous, and 27.5 for dry substances. Notably, the formation of dry membranous substances warrants special attention as they potentially compromise the quality of life of older patients during tube feeding and increase the burden on caregivers during oral care procedures. Therefore, maintaining oral wetness above the specified cut‐off value through consistent oral care and moisturization is essential for improving patient outcomes.

## Author Contributions

Hironao Asahina contributed to the conceptualization of the study design, data collection, data analysis, interpretation, and manuscript drafting. Yoshiyuki Okada was involved in data acquisition and interpretation. Ryo Nishino contributed to data acquisition. Kahoru Todoroki and Kohei Matsumura played an important role in diagnosing oral membranous substances. Yusuke Yamagami contributed reagents, materials, and analytical tools. Tadashi Ogasawara provided research advice, guidance, and manuscript revisions. All authors reviewed and approved the final version of the manuscript to be published and agreed to be accountable for all aspects of the work, ensuring that questions related to the accuracy or integrity of the research are appropriately investigated and resolved.

## Ethics Statement

This study was approved by the Ethics Committee of Hiroshima University (approval number: E2022‐0243).

## Conflicts of Interest

The authors declare no conflicts of interest.

## Supporting information




**Figure S1**: Flowchart of participant recruitment

**Figure S2**: Correlation between the morphology of membranous substances (morphology: 1, none; 2, mucous substances; 3, viscous substances; 4, dry membranous substances) and oral wetness. Results of the combined analysis of all data at each elapsed time are shown (*n* = 90).

**Figure S3**: Correlation between the morphologies of membranous substances (morphology: 1, none; 2, mucous substances; 3, viscous substances; 4, dry membranous substances) and elapsed time. Results of the combined analysis of all data at each time point are shown (*n* = 90).

## Data Availability

Research data are not shared.
