# Supplementary figures and images for: Relationship Between the Morphology of Oral Membranous Substances and Oral Wetness in Older Individuals Undergoing Long‐Term Tube Feeding in Japan
Source: Spec Care Dentist. 2025 Aug 18;45(4):e70078. doi: 10.1111/scd.70078 (PMC12361872; doi:10.1111/scd.70078)

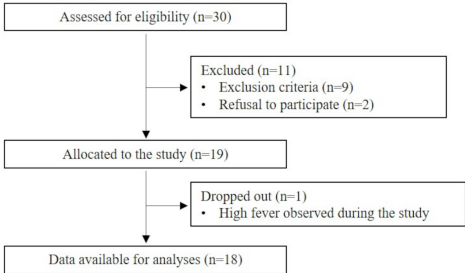

Supplement: Supplementary file 1 — Figure S1: Flowchart of participant recruitment [file SCD-45-0-s001.pdf]

Morphologies of the membranous substances

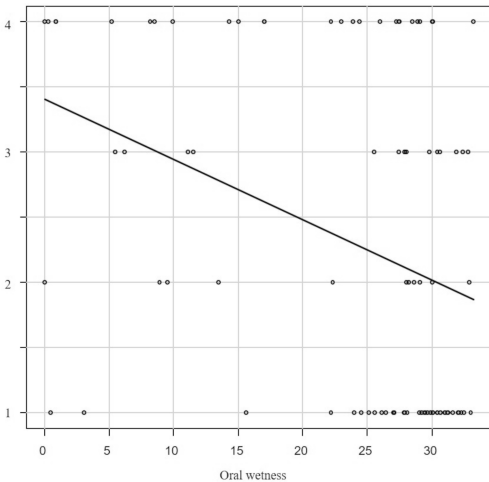

Supplement: Supplementary file 2 — Figure S2: Correlation between the morphology of membranous substances (morphology: 1, none; 2, mucous substances; 3, viscous substances; 4, dry membranous substances) and oral wetness. Results of the combined analysis of all data at each elapsed time are shown (n = 90). [file SCD-45-0-s002.pdf]

Morphologies of the membranous substances

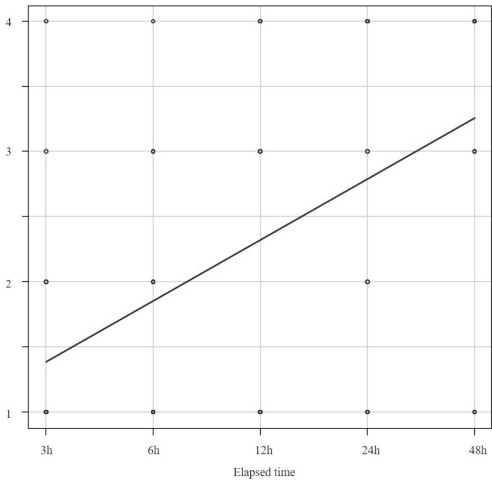

Supplement: Supplementary file 3 — Figure S3: Correlation between the morphologies of membranous substances (morphology: 1, none; 2, mucous substances; 3, viscous substances; 4, dry membranous substances) and elapsed time. Results of the combined analysis of all data at each time point are shown (n = 90). [file SCD-45-0-s003.pdf]
